# Hospital Admissions Due to Ischemic Heart Diseases and Prescriptions of Cardiovascular Diseases Medications in England and Wales in the Past Two Decades

**DOI:** 10.3390/ijerph18137041

**Published:** 2021-07-01

**Authors:** Sara Ibrahim Hemmo, Abdallah Y. Naser, Hassan Alwafi, Munthir M. Mansour, Abeer F. R. Alanazi, Zahraa Jalal, Zahra Khalil Alsairafi, Vibhu Paudyal, Esra’a Alomari, Hamzeh Al-Momani, Emad M. Salawati, Mohammed Samannodi, Mohammad S. Dairi, Abdel Qader Al Bawab, Moaath K. Mustafa Ali, Saqer Alkharabsheh

**Affiliations:** 1 Department of Applied Pharmaceutical Sciences and Clinical Pharmacy, Faculty of Pharmacy, Isra University, Amman 11622, Jordan; ac1012@iu.edu.jo (S.I.H.); esraa.alomari@iu.edu.jo (E.A.); 2 Faculty of Medicine, Umm Alqura University, Mecca 21514, Saudi Arabia; hassan.alwafi.16@ucl.ac.uk (H.A.); samannodi@gmail.com (M.S.); msdairi@uqu.edu.sa (M.S.D.); 3 Department of Internal Medicine, University of Arkansas for Medical Center, Little Rock, AR 72205, USA; drmunthir86@gmail.com; 4 Department of Pharmaceutical and Biological Sciences, UCL School of Pharmacy, London WC1E 6BT, UK; abeer.alanazi.18@ucl.ac.uk; 5 School of Pharmacy, Institute of Clinical Sciences, University of Birmingham, Birmingham B15 2TT, UK; z.jalal@bham.ac.uk (Z.J.); V.Paudyal@bham.ac.uk (V.P.); 6 Department of Pharmacy Practice, Kuwait University, Kuwait 12037, Kuwait; zahra.alsairafi@ku.edu.kw; 7 Department of Pediatric, School of Medicine, Hashemite University, Zarqa 13133, Jordan; hamzehmomani82@hotmail.com; 8 Family Medicine Department, Faculty of Medicine, King Abdulaziz University, Jeddah 22254, Saudi Arabia; esalawati@kau.edu.sa; 9 Faculty of Pharmacy, Al-Zaytoonah University of Jordan, Amman 11733, Jordan; abdelqader.albawab@zuj.edu.jo; 10 Greenebaum Comprehensive Cancer Center, University of Maryland, Baltimore, MD 20742, USA; moaath_mustafa@yahoo.com; 11 Heart and Vascular Institute, Cleveland Clinic, Cleveland, OH 44195, USA; saqarkh@gmail.com

**Keywords:** IHD, admission rate, IHD medication, trend, IHD rate, IHD medication rate

## Abstract

Objectives: The aim of this study was to explore the trend of ischemic heart disease (IHD) admission and the prescriptions of IHD medications in England and Wales. Methods: A secular trends study was conducted during the period of 1999 to 2019. We extracted hospital admission data for patients from all age groups from the Hospital Episode Statistics database in England and the Patient Episode Database for Wales. Prescriptions of IHD medications were extracted from the Prescription Cost Analysis database from 2004 to 2019. The chi-squared test was used to assess the difference between the admission rates and the difference between IHD medication prescription rates. The trends in IHD-related hospital admission and IHD-related medication prescription were assessed using a Poisson model. The correlation between hospital admissions for IHD and its IHD medication-related prescriptions was assessed using the Pearson correlation coefficient. Results: Our study detected a significant increase in the rate of cardiovascular disease (CVD) medication prescriptions in England and Wales, representing a rise in the CVD medications prescription rate of 41.8% (from 539,334.95 (95% CI = 539,286.30–539,383.59) in 2004 to 764,584.55 (95% CI = 764,545.55–764,623.56) in 2019 prescriptions per 100,000 persons), with a mean increase of 2.8% per year during the past 15 years. This increase was connected with a reduction in the IHD hospital admission rate by 15.4% (from 838.50 (95% CI = 836.05–840.94) in 2004 to 709.78 (95% CI = 707.65–711.92) in 2019 per 100,000 persons, trend test, *p* < 0.01), with a mean decrease of 1.02% per year during the past 15 years and by 5% (from 747.43 (95% CI = 745.09–749.77) in 1999 to 709.78 (95% CI = 707.65–711.92) in 2019 per 100,000 persons, trend test, *p* < 0.01) with a mean decrease of 0.25% per year during the past two decades in England and Wales. Conclusion: The rate of hospitalisation due to IHD has decreased in England and Wales during the past two decades. Hospitalisation due to IHD was strongly and negatively correlated with the increase in the rates of dispensing of IHD-related medications. Other factors contributing to this decline could be the increase in controlling IHD risk factors during the past few years. Future studies exploring other risk factors that are associated with IHD hospitalisation are warranted.

## 1. Introduction

Cardiovascular diseases are the leading cause of mortality globally, accounting for an estimated 17.9 million deaths in 2016 [1]. Among cardiovascular diseases, ischemic heart diseases (IHD) is the leading cause of death worldwide [2]. In 2017, IHD affected about 126 million individuals, with an estimated prevalence of 1655 per 100,000 [2,3]. The prevalence of IHD is projected to increase to 1845 per 100,000 by 2030 [3]. However, the incidence rate of IHD is decreasing worldwide [2]. The discrepancy between IHD prevalence and incidence is likely explained by improved treatment of patients with IHD, hence the improved survival rate. The increasing prevalence of IHD may increase the pool of patients who experience decompensations and the need for hospitalisation.

Like in many other countries, IHD is the number one cause of mortality in the United Kingdom (UK). In 2014, 10% of female mortality and 15% of male mortality was due to IHD, contributing to 69,000 deaths [4]. In the UK, IHD is the leading cause of death among males from all age groups since 2001 until 2018 [5]. Bhatnagar et al. reported that IHD inpatient events were two times less common among women than men, estimating 1.4% in women and 3.4% in men of all inpatient episodes in the UK. In the National Health Service (NHS) hospitals in the UK, there were more than 491,000 inpatient events of IHD in 2013–2014, about 401,000 in England, and more than 25,000 in Wales; the gender diversity was likewise noticeable in all UK nations. Between 1974 and 2013 in the UK, IHD mortality rates decreased by 73% [4].

Despite the fact that females usually have a lower probability of developing CVDs compared to males, they have poor prognosis and a higher rate of mortality following an acute cardiovascular episode [6]. Cardiovascular disease risk in females is commonly underestimated due to the misunderstanding that they are relatively more protected compared to males against CVD [7]. This could have led to approaching less aggressive treatment strategies with them [7]. Due to the gender-related difference between males and females in terms of the development of CVD, this increased the need for a therapeutic approach to CVD that is gender-specific [8,9]. Therefore, exploring the role of gender in terms of hospitalisation rate related to CVD is important for the management and prevention of CVD.

Although the death rate from cardiovascular disease (CVD) is decreasing, it remains the principal cause of death in the world [10,11]. This study aims to investigate the trends in IHD-related hospital admissions (IRHAs) and the prescription of cardiovascular disease (CVD)-related medications in England and Wales in the past decade between 1999 and 2019. Additionally, we aim to study the correlation between them. Identifying patterns of hospitalisation related to specific IHD types are helpful from a public health and prevention point of view. Additionally, exploring gender- and age-based differences in IHD-related hospital admission rates will help in identifying which demographic group would benefit from future prevention/public health interventions.

## 2. Methods

### 2.1. Study Sources and the Population

We conducted a secular trends study using publicly available data extracted from the Hospital Episode Statistics (HES) database in England [12] and the Patient Episode Database for Wales (PEDW) for the period between April 1999 and April 2019 [13]. The HES and PEDW databases contain hospital admission data for patients with IHD from all age groups. The age groups are subdivided into four categories: below 15 years, 15–59 years, 60–74 years, and 75 years and more. We identified IHD hospital admissions using the tenth version of the International Statistical Classification of Diseases (ICD) system [14]. All diagnostic codes for IHD (I20–I25) were used to identify all hospital admissions related to various types of IHD in England and Wales. HES and PEDW data are checked regularly to ensure their validity and accuracy [12,15]. To calculate the annual hospital admission rate for IHD, we collected mid-year population data for the period between 1999 and 2019 from the Office for National Statistics (ONS) [16].

Cardiovascular disease-related medication prescription data in England and Wales were extracted from the Prescription Cost Analysis database for the available period of April 2004–March 2019 [17]. The British National Formulary (BNF) therapeutic classification system is used to report the PCA database data medication categories [18]. Prescription data became available in England from 2004 and in Wales from 2000. Cardiovascular disease medications were identified using the cardiovascular system chapter in the BNF (Chapter 2). Specific cardiovascular medications recommended for the management of each ischemic heart disease sub-class (I20–I25) were identified using National Institute for Health and Care Excellence (NICE) guidelines [19].

### 2.2. Statistical Analysis

The rates of hospital admission with their 95% confidence intervals (CIs) were calculated using the number of IHD admissions for each age group divided by the mid-year population of the same age group of the same year. IHD medication prescription rates were calculated using the number of IHD medication prescriptions divided by the total mid-year population during the same year. The chi-squared test was used to assess the difference between the admission rates in 1999 and 2019 and the difference between IHD medication prescription rates in 2004 and 2019. The trends in IHD-related hospital admission and IHD-related medication prescription were assessed using a Poisson model. The correlation between hospital admissions for IHD and its IHD medication-related prescriptions was assessed for the duration between 2004 and 2019 using the Pearson correlation coefficient. Only a subset of IHD-related medications was selected for correlation analysis with IHD-related disease based on clinical relation/indication. A two-sided *p* < 0.05 was considered statistically significant. All analyses were performed using SPSS software version 25 (IBM Corp, Armonk, NY, USA).

## 3. Results

A total of 8,598,641 IRHAs were recorded in England and Wales during the duration of the study. The absolute number of annual IRHAs for different causes increased from 389,712 in 1999 to 421,893 in 2019. However, the rate of annual IRHAs decreased by 5.0% (from 747.43 (95% CI = 745.09–749.77) in 1999 to 709.78 (95% CI = 707.65–711.92) in 2019 per 100,000 persons, trend test, *p* < 0.01). The most common IHD-related hospital admission reasons were chronic IHD, acute myocardial infarction, and angina pectoris (Table 1).

**Figure 1:**
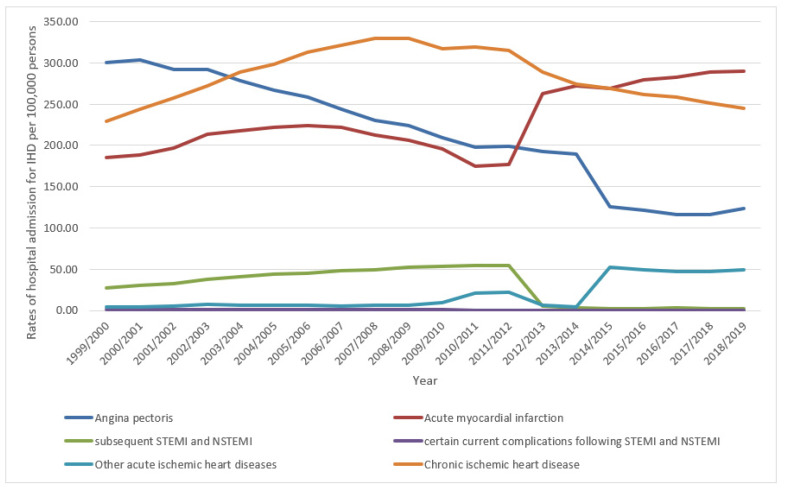
Hospital admission rates due to IHD in England and Wales stratified by type between 1999 and 2019.

Over the last two decades, the hospitalisation rates for the other acute IHDs category, acute myocardial infarction, and chronic IHD increased by 12-fold (1175.0%), 56.3%, and 6.8%, respectively. On the contrary, the rate of hospital admission due to subsequent STEMI and NSTEMI, certain current complications following STEMI and NSTEMI, and angina pectoris decreased by 90.8%, 63.5%, and 59.1%, respectively (Figure 1).

**Figure 2:**
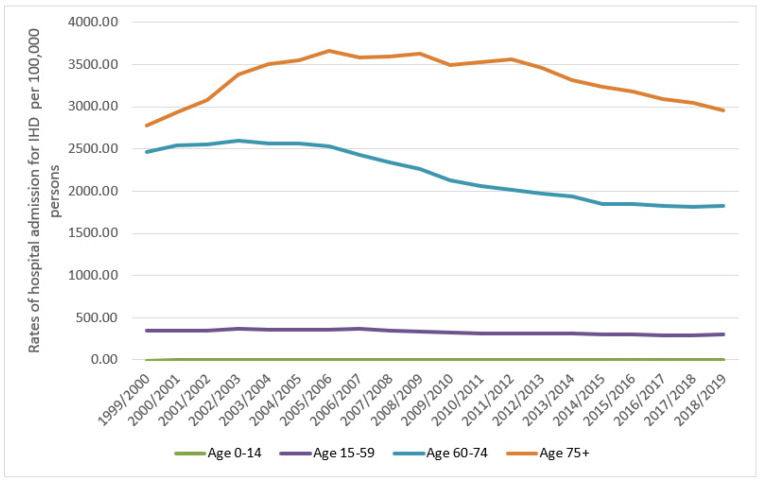
Rates of hospital admission for all IHD in England and Wales stratified by age group.

Regarding age group differences in IRHA, the 60–74-year age group contributed to 40.5% of the total number of admissions, followed by those aged 75 years and above at 34%, then 15–59 years at 25.5%. The IHD-related hospital admission rate among patients aged below 15 years increased by 114.5% (from 0.28 (95% CI = 0.18–0.39) in 1999 to 0.61 (95% CI = 0.46–0.75) in 2019 per 100,000 persons, trend test, *p* < 0.001). The IHD-related hospital admission rate among patients aged 15–59 years decreased by 14.8% (from 351.07 (95% CI = 349.00–353.14) in 1999 to 299.23 (95% CI = 297.40–301.05) in 2019 per 100,000 persons, trend test, *p* < 0.001). The IHD-related hospital admission rate among patients aged 60–74 years decreased by 25.9% (from 2462.48 (95% CI = 2450.95–2474.02) in 1999 to 1825.19 (95% CI = 1816.56–1833.82) in 2019 per 100,000 persons, trend test, *p* < 0.001). The IHD hospital admission rate among patients aged 75 years and above increased by 6.5% (from 2777.18 (95% CI = 2760.90–2793.46) in 1999 to 2957.84 (95% CI = 2943.11–2972.58) in 2019 per 100,000 persons, trend test, *p* < 0.001) (Figure 2). Between 1999 and 2005, IHD- related hospital admission rates among patients aged 75 years and above increased by 27.9%. This was followed by a period of relative plateauing (2005 to 2011), during which the IHD-related hospital admission rate decreased by 2.7%. In the period 2011–2019, there was a rapid decrease in the IHD hospital admission rate by 17.0%.

**Figure 3:**
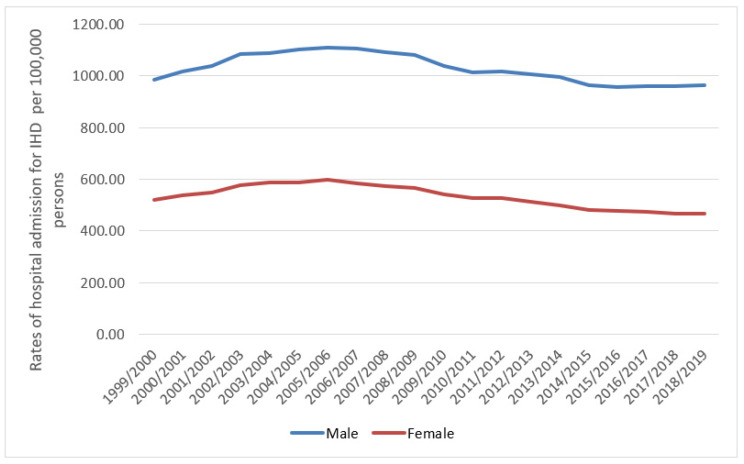
Rates of hospital admission for all IHD in England and Wales stratified by gender.

Males contributed 65.1% of the total number of IRHA, accounting for 5,601,491 hospital admission episodes, with a mean of 280,074 per year. The IHD hospital admission rate of females decreased by 10.4% (from 521.06 (95% CI = 518.33–523.79) in 1999 to 466.87 (95% CI = 464.43–469.31) in 2019 per 100,000 persons, trend test, *p* < 0.05). The IHD-related hospital admission rate of males decreased by 2.3% (from 985.39 (95% CI = 981.55–989.23) in 1999 to 962.52 (95% CI = 958.99–966.05) in 2019 per 100,000 persons, trend test, *p* = 0.24) (Figure 3).

### 3.1. IHD Hospital Admission Rate by Gender

**Figure 4:**
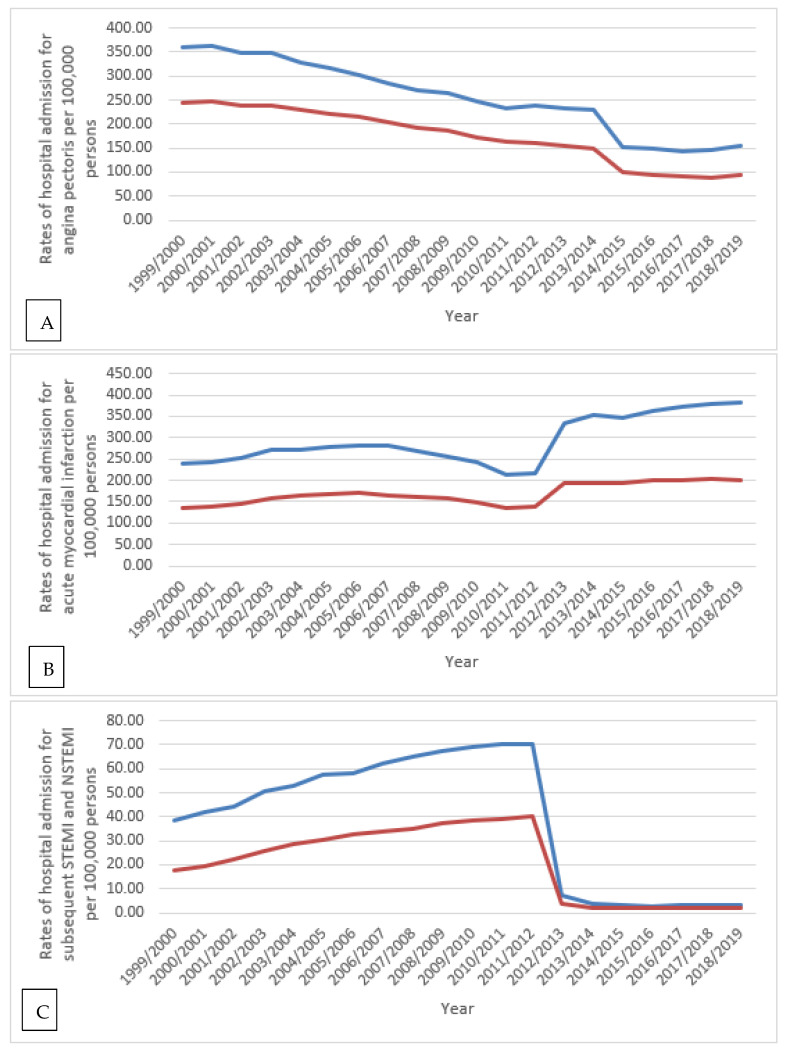

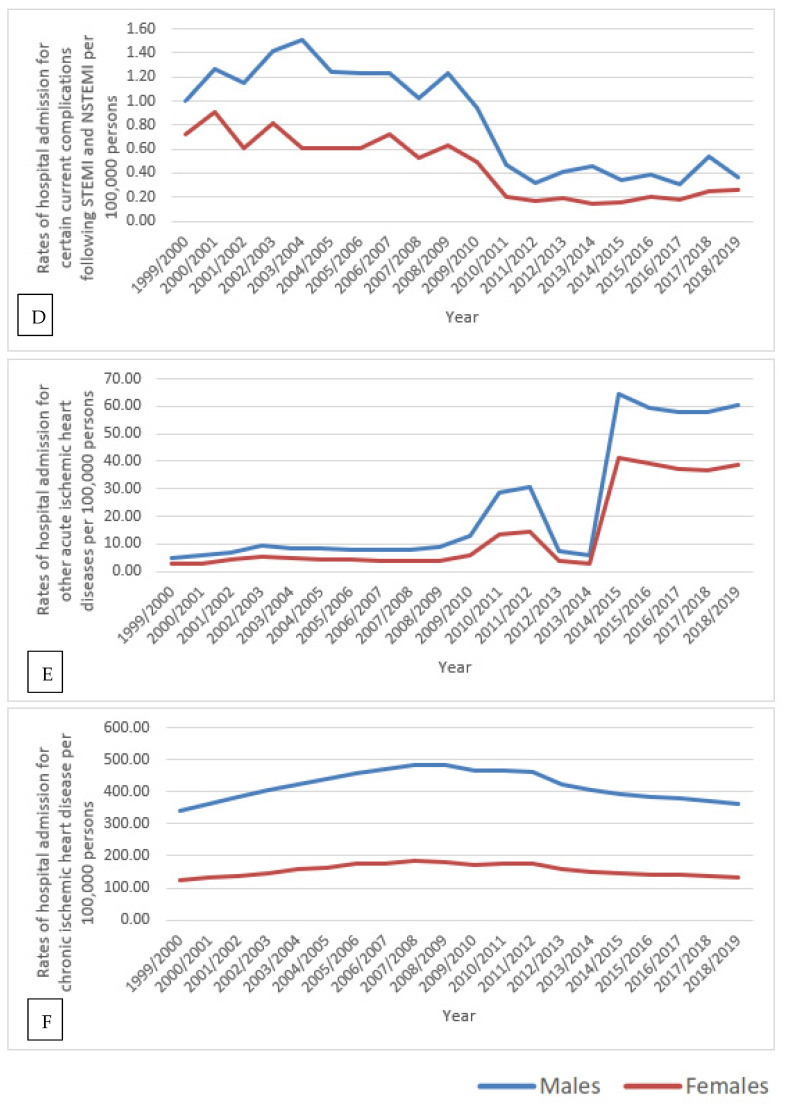
Hospital admission rates for IHD in England and Wales stratified by gender. (**A**): rate of hospital admission for angina pectoris, (**B**): rate of hospital admission for acute myocardial infarction, (**C**): rate of hospital admission for subsequent STEMI and NSTEMI, (**D**): rate of hospital admission for certain current complications following STEMI and NSTEMI, (**E**): rate of hospital admission for other acute ischemic heart diseases, (**F**): rate of hospital admission for chronic ischemic heart diseases.

IHD-related hospital admission rates for all types were higher among males compared to females (*p* < 0.05) (Figure 4).

### 3.2. IHD Hospital Admission Rate by Age Group

**Figure 5:**
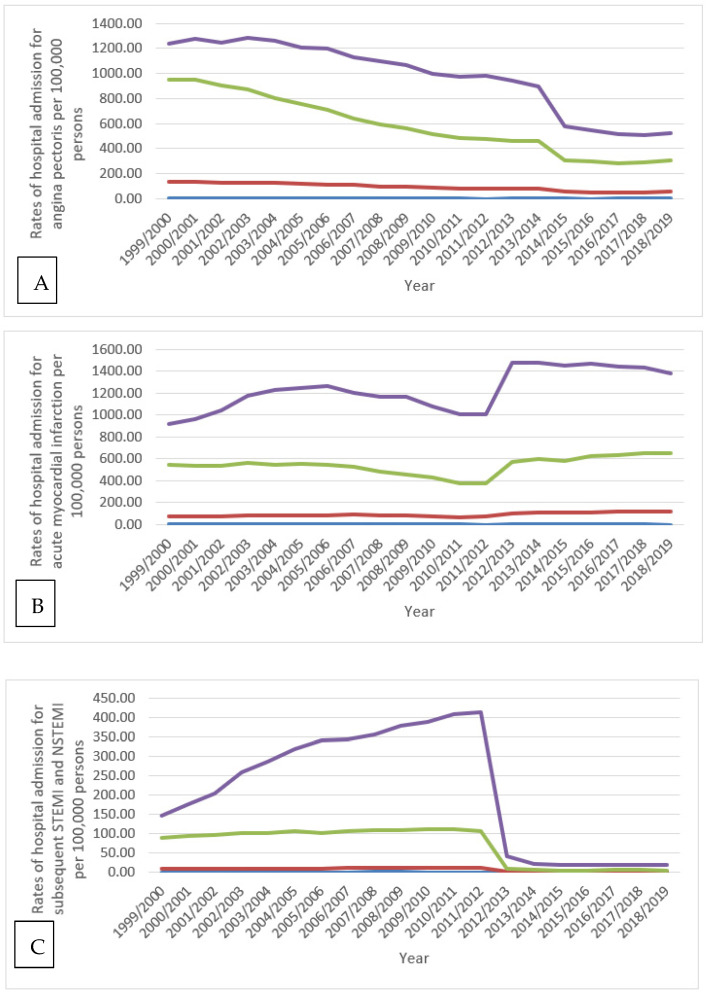

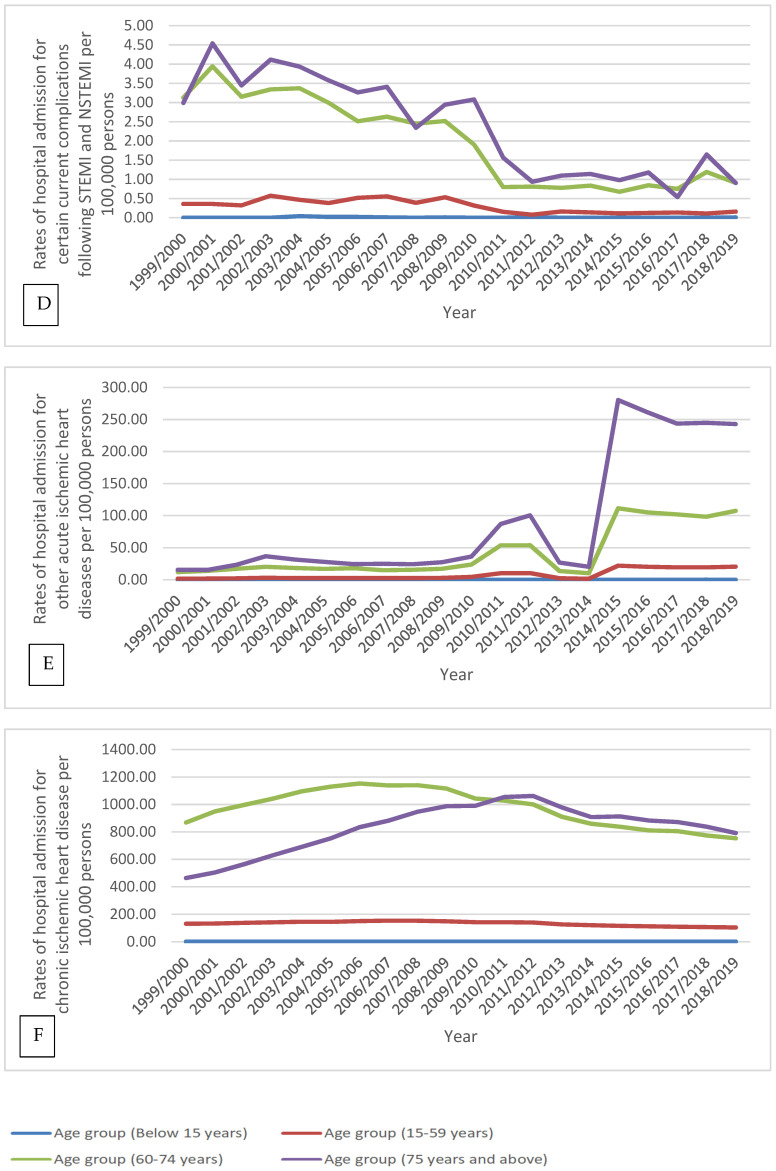
Hospital admission rates for IHD in England and Wales stratified by age group. (**A**) rate of hospital admission for angina pectoris, (**B**) rate of hospital admission for acute myocardial infarction, (**C**) rate of hospital admission for subsequent STEMI and NSTEMI, (**D**) rate of hospital admission for certain current complications following STEMI and NSTEMI, (**E**) rate of hospital admission for other acute ischemic heart diseases, (**F**) rate of hospital admission for chronic ischemic heart diseases.

The rate of certain current complications following STEMI and NSTEMI was higher among the 60–74-year age group than those aged 75 years and above during the years 1999/2000, 2007/2008, and 2016/2017. Furthermore, the rate of chronic IHD was higher among the 60–74-year age group than the 75-year age group from 1999 to 2010, after which the rate of chronic IHD became higher again in those aged 75 years and older from 2010 to 2019 (Figure 5).

### 3.3. Cardiovascular Disease-Related Medication Prescriptions.

**Figure 6:**
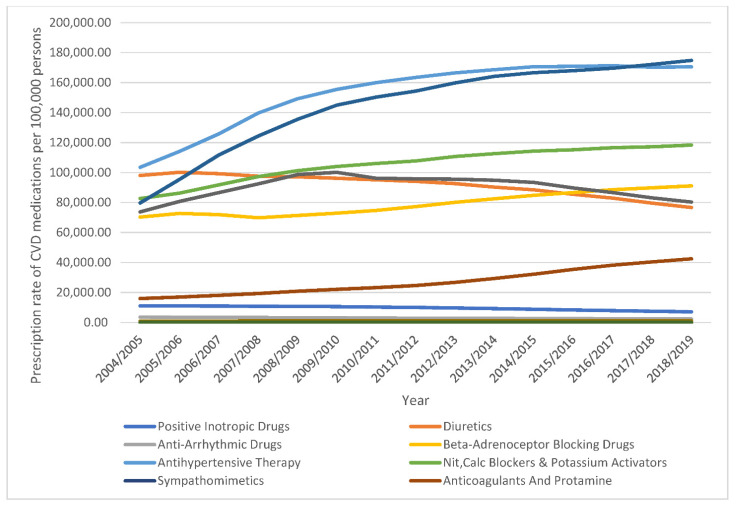
Prescription rates of CVD medications in England and Wales between 2004 and 2019.

The absolute number of CVD-related medication prescriptions dispensed annually in England and Wales increased by 59.7% (from 217,567,178 in 2004 to 347,541,856 in 2019). CVD medication prescription rates increased by 41.8% (from 539,334.95 (95% CI = 539,286.30–539,383.59) in 2004 to 764,584.55 (95% CI = 764,545.55–764,623.56) in 2019, prescriptions per 100,000 persons, trend test, *p* < 0.001) (Table 2) (Figure 6) (detailed CVD-related medication prescription rates per sub-class are available in the Supplementary Materials section).

### 3.4. Correlation between IHD Hospitalisation and the Prescription of CVD Medications

Table 3 shows the correlation coefficient between IHD-related hospital admission rates and specific IHD medication prescription rates between 2004 and 2019.

#### 3.4.1. Angina Pectoris

A strong negative correlation was found between the prescription of beta-adrenoceptor blocking drugs, antihypertensive therapy, nitrates, calcium channel blockers and potassium channel activators, lipid-regulating drugs, and hospital admission rates for angina pectoris (*p* < 0.01).

#### 3.4.2. Acute Myocardial Infarction

A strong negative correlation was found between the prescription of positive inotropic drugs, diuretics, anti-arrhythmic drugs, and hospital admission rates for acute myocardial infarction (*p* < 0.05).

#### 3.4.3. Subsequent ST Elevation (STEMI) and Non-ST Elevation (NSTEMI) Myocardial Infarction

A negative correlation was found between the prescription of beta-adrenoceptor blocking drugs, antihypertensive therapy, nitrates, calcium channel blockers and potassium channel activators, anti-fibrinolytic drugs and haemostatics, lipid-regulating drugs, and hospital admission rates for subsequent ST elevation (STEMI) and non-ST elevation (NSTEMI) myocardial infarction (*p* < 0.05).

#### 3.4.4. Certain Current Complications Following ST Elevation (STEMI) and Non-ST Elevation (NSTEMI) Myocardial Infarction (Within the 28-Day Period)

A strong negative correlation was found between the prescribing of beta-adrenoceptor blocking drugs, antihypertensive therapy, nitrates, calcium channel blockers and potassium channel activators, anti-fibrinolytic drugs and haemostatics, lipid-regulating drugs, and hospital admission rates for certain current complications following ST elevation (STEMI) and non-ST elevation (NSTEMI) myocardial infarction (within the 28-day period) (*p* < 0.01).

#### 3.4.5. Chronic Ischemic Heart Disease

A negative correlation was found between the prescribing of beta-adrenoceptor blocking drugs, antihypertensive therapy, nitrates, calcium channel blockers and potassium channel activators, lipid-regulating drugs, and hospital admission rates for chronic ischemic heart disease (*p* < 0.05).

## 4. Discussion

Our study showed that CVD medications prescribed in England and Wales increased significantly by 41.8% between 2004 and 2019. The mean increase was 2.8% per year during the past 15 years. This increase was concomitant with a 15.4% reduction in the IHD hospital admission rate, with a mean decrease of 1.02% annually during the past 15 years, and by 5% between 1999 and 2019, with a mean decrease of 0.25% annually.

Congruent with our results, previous studies found an increase in the dispensing of IHD medications in England and Wales [4,20]. CVD drug prescription increased in England between 1999 and 2014 by 74% and in Wales between 2005 and 2014 by 23% [20]. The dispensing of CVD medications in England in 2014 was approximately seven times more than in 1981 [4]. In Wales, the dispensing of CVD medications increased by 5.5 million between 2005 and 2014 [4].

Prior studies found that the hospitalisation rate due to IHD decreased during the past years in the UK [4,16]. Between 1999 and 2007, the prevalence rate of IHD and IHD hospitalisations declined by 3–4% annually in the UK [21]. At the same time, our study found that the rate of IHD hospitalisations in England and Wales decreased by 0.25% per year between 1999 and 2019. The difference is likely due to the variation in study periods included, as well as the geographical area examined.

Our study found that, during the past two decades, the total number of hospitalisations due to IHD among males was almost twice that of females (65.1% males versus 34.9% females), which is consistent with a previous study [4]. The higher rate in males is likely explained by the higher prevalence and incidence of IHD among males [3,22,23]. In a 2015 study by Bhatnagar et al., the number of IRHAs decreased by 4% among men and 11% among women between 2005/2006 and 2013/2014 in England [4]. This confirms the findings of our study, whereby IRHAs decreased by 11.4% among females (from 163,634 in 2005/2006 to 144,914 in 2013/2014) and by 3.5% among males (from 291,989 in 2005/2006 to 281,740 in 2013/2014). Multiple previous studies have reported that females are more prone to CVDs at an older age compared to males [6,24]. This could be due the underestimation of their risk of developing CVD compared to males [7], which might lead to approaching less aggressive treatment strategies with them [7]. Despite that, in our study, we found that the hospitalisation rate for IHDs was higher among males compared to females for most types of IHDs. This could be attributed to their lifestyle, including physical inactivity and poor diet [25]. At the same time, there are other risk factors (psychosocial risk factors) that affect the traditional risk factors and increase males’ risk of developing CVDs and its associated complications that could lead ultimately to the need for urgent medical assistance and hospitalisation. These include excess aggression, impatience, and competitiveness, besides more recent type of personalities that are linked with depression, anxiety, and low socioeconomic status [26]. This increased risk was proposed to be mainly driven by mechanisms disturbed autonomic and neuroendocrine regulation [27,28].

The prevalence and incidence of IHD increase with aging [22,23,29,30]. The higher prevalence and incidence of IHD explains the higher percentage of IRHA in the elderly age group compared to younger age groups. A previous study in the Netherlands evaluating hospitalisations trends due to IHD found that the rate of hospitalisation decreased in patients <75 years and increased in patients of a very advanced age (≥85 years) between 1998 and 2007 [31]. Our study found that the IHD-related hospital admission rate decreased in patients <75 years and increased in patients of a very advanced age (≥75 years) between 1999 and 2007.

The same study found that the hospitalisation rates for chronic IHD decreased slightly in patients <65 years and increased in patients ≥65 years between 1998 and 2007. This is contrary to our study, which showed that the chronic IHD-related hospital admission rates increased for all age groups between 1999 and 2007 (Figure 5). In the Netherlands, the hospitalisation rates for acute myocardial infarction decreased among patients <95 years and increased slightly among patients ≥95 years between 1998 and 2007. On the other hand, we found that the hospitalisation rates for acute myocardial infarction rose in <15 years, 15–59 years, and ≥75-year age groups and decreased in 60–74-year age group between 1999 and 2007 (Figure 5).

IHD hospitalisation rates decreased in the Netherlands in the last few years, with a significant decrease in acute myocardial infarction hospital admission rates, while chronic IHD hospitalisation rates increased [31]. Regarding the recent decrease in hospitalisation rates for IHD, the same result was seen in our study in England and Wales. Moreover, chronic IHD hospitalisation trends were similar in the Netherlands and England/Wales. On the other hand, the acute myocardial infarction hospital admission rate increased in England/Wales, but decreased in the Netherlands (Figure 1).

The prevalence rate of angina pectoris in England dropped from 4.8% to 3.2% in males and 3.4% to 2.1% in females in the years 2003 and 2017, respectively [32]. This decrease in prevalence likely explains the decrease in angina pectoris-related hospitalisations in males and females, as shown in our study (Figure 4). During the past two decades in England and Wales, the increase in the prescription rates of nitrates, calcium channel blockers, potassium activators, and beta-adrenoceptor blocking drugs may have improved angina pectoris management in the outpatient setting and subsequently dropped angina pectoris-related hospitalisation rates (Figure 1, Figure 6, and Table 2).

In England, cigarette smoking among adults decreased from 27% in 1993 to 17% in 2017. Additionally, the percentage of children (aged 8 to 15) who had ever smoked declined from 19% to 5% in the same period [29]. Between 2011 and 2017 in England, alcohol consumption decreased among adults and children [29]. The progressive drop in smoking and alcohol consumption has likely contributed to the decrease in IHD-related hospital admission rates seen nowadays.

In England, the percentage of adults diagnosed with hypercholesterolemia decreased from 67% in 1998 to 48% in 2017 [29]. The significant increase in the prescription of lipid-regulating drugs led to improved control of hyperlipidemia (Figure 6 and Table 2). Because hyperlipidemia is a significant risk factor for IHD, we hypothesise that increased use of lipid-regulating drugs has improved IHD outcomes and subsequently decreased the IHD-related hospital admission rate.

The proportion of adults with untreated hypertension decreased from 16% in 2003 to 11% in 2017 in females and from 20% to 12% in males [29]. As shown in our study, the prescription of antihypertensive medications increased by 65%; this likely decreased IHD incidence and associated morbidity and the subsequent IHD-related hospital admission rate.

A previous study infers a parallel rise in rates of dysglycaemia hospital admission and rates of dispensing antidiabetic medication in England and Wales between 1999 and 2016 [33]. In England (1994–2017), the percentage of adults reporting doctor-diagnosed diabetes rose from 2% to 5% among females and from 3% to 8% among males [29].

Consistent with other studies, the use of anti-anginal medications, such as beta-blockers, calcium channel blockers, and disease-modifying medications like anti-platelets, and lipid-lowering medications, are negatively correlated with IHD-related hospital admission rates. These medications are mainly maintenance therapy and have been shown to improve symptoms and prolong survival in many studies [34,35,36,37,38]. In acute myocardial infarction, there is a positive correlation with the prescription of nitrates, beta-blockers, and lipid-lowering medications, which is likely attributed to the increased utilisation of these medications during hospitalisations. We noted a positive correlation of the use of inotropic medications and diuretics among almost all types of IHD, and this is likely a reflection of the increased prevalence of heart failure diagnosis, whether systolic or diastolic, following IHD diagnosis/admissions. In our study, some CVD medication classes showed a positive correlation with the IHDs hospitalisation rate; these classes are mainly used in the inpatient settings as emergency treatment. Reasons that could also contribute to the increased prescription of CVD medicines include: updates in practice guidelines, a safer profile, and advancement in drug development and diagnosis of the disease. A previous large cohort study in the UK reported that, between 2002 and 2014, despite a moderate decline in the standardised incidence of heart failure, the burden of heart failure in the UK increased [39].

Previous studies revealed that obesity is an independent predictor of the future risk of hospital admissions due to IHD. For example, Murphey et al. predicted an additional 14 admissions per 100 men and women over the next 20 years if the prevalence of obesity continues to rise [40]. Although studies have revealed an increasing prevalence of obesity worldwide [41], recent evidence revealed a levelling off in the prevalence of obesity in certain countries, including England, since the year 1999 when compared with the earlier trend [42,43]. In the UK, there is an increased government interest in healthy lifestyles. The government announced its ambition via the Healthy Weight, Healthy Lives programme and the Choosing Health: Making Healthy Choices Easier programme to be the first country to implement public measures and prevention strategies to tackle adult obesity and a sedentary lifestyle over the coming decades [10,44]. We believe that the gradual decrease in the rate of obesity, together with the increased rate of prescriptions for IHD medications, may have contributed to the decrease in hospitalisation rates due to IHD.

Over the past two decades, many factors may have influenced the trend of admission rates due to IHD. For instance, the introduction of coronary computed tomography angiography (CCTA) and coronary artery calcium score (CACS) and their wide use since 2011 as an anatomical diagnostic tool has completely changed the diagnostic approach of coronary artery disease (CAD). Most major adverse cardiac events (MACEs) requiring hospitalisation take place in non-obstructive plaques, particularly in women [45], and the use of an anatomical approach to diagnosing CAD has led to earlier diagnosis of CAD than the conventional functional approach. The ability to detect non-obstructive plaques on CCTA, even before the onset of symptoms using CACS [46], has led to an incremental increase in the rate of prescribed cardioprotective medications over the last decade, which subsequently decreased the incidence of MACE, IHD, and revascularisation [47,48]. Other factors to consider include the introduction of drug-eluting stents, which improved outcomes after percutaneous coronary intervention (PCI) and decreased the risk for restenosis and MACEs compared to conventional stents [49]. Furthermore, it is worth mentioning that the introduction of the newer anti-platelet agents, e.g., Ticagrelor, have also improved the outcomes compared to the old generation drugs, e.g., clopidogrel [50]. The advancement of PCI techniques (intravascular brachytherapy) and functional flow reserve have significantly helped to improve IHD-related symptoms and, potentially, IRHAs [51]. Concerning the effectiveness of cardiac rehabilitation programmes in decreasing the risk of hospital admission, a recent study that was conducted on 249 patients with a recent history of acute coronary syndrome has reported that a rehabilitation programme did not show any additional functional improvement to the patients [52].

IHD prevalence continues to increase despite the decreasing IHD incidence. This is likely due to the implementation of public health measures and the recent efforts to tackle cardiovascular risk factors, coupled with the increased cardiovascular medication prescription rate [53]. We observed a reduction in IRHAs despite the increasing prevalence; this is likely due to the unfolding of highly intensive therapy, such as newer anti-platelet agents and the wide use of high-intensity statins, leading to plaque stabilisation and subsequently the prevention of MACEs and related hospitalisations [54]. Another cause of the increased prevalence, despite the decreased incidence, is the increased life expectancy overall in the UK, which is a reflection of the public health measures taken to improve population risk factors. Understanding the trends of IHD admissions and the correlated risk factors and variables will help to direct public health campaigns to tackle significant IHD risk factors.

### Strengths and Limitations

To the best of our knowledge, this is the first study to explore trends in the rates of IHD-related hospitalisation and the prescription of IHD-related medications worldwide and in England and Wales without restricting the study to specific inclusion/exclusion criteria. Our study provided detailed hospital admission rates for all types of IHD stratified by age and gender, giving a comprehensive description of the hospitalisation profile for this group of patients over 20 years. Our study has several limitations. Since it is a secular trends study, it lacks information at the individual levels. Having a family history of CVD, personal lifestyle, overweightness/obesity, alterations in lipid metabolism, and lack of physical activity are also variables that can impact the incidence or prevalence of IHD/CVD. Because of the study’s nature, the correlations between IHD-related hospital admission rates and IHD-related medication prescription rates cannot be confirmed. Innate to ecologic studies, they cannot establish causality or associations. Identified temporal variability (the increase in the rate of hospitalisation due to acute myocardial infarction starting from the year 2012/2013 and the decrease in the rate of hospitalisation due to STEMI/NSTEMI) could be related to potentially non-medical causes, such as updated coding guidelines. These artificial changes may introduce temporal correlation among diagnoses inferred from routine data, violating the assumptions of frequently used statistical methods [55].

In correlation analysis, there is a lack of information on the temporal relation between the drugs and associated IHD complications. Moreover, data are not available at the individual level. Hence, statistically significant results do not necessarily imply causality. They may indicate possible causality, reverse causality, or confounded association. Accounting for confounding association remains crucial in interpreting the analysis, as the care of patients with IHD has improved in multiple ways, and many innovations have been introduced during the last 20 years. Moreover, many medications are used after an IHD complication occurs, resulting in a positive association, which is defined as reverse causality. When looking at the overall changes during the study period in admission and prescription rates without considering the weight of group (the CVD medications (sympathomimetic and local sclerosants) and IHD-specific type STEMI and NSTEMI contribute to only 0.1% of the total number of admissions) from the total number of prescriptions/admissions, overweighting may lead to overestimated rates, which could affect the interpretation of the study findings. Admission data include admission and readmission at the same time. Therefore, our admission rate estimates could be overestimated, as some proportion of them could be drug-use related re-admission (due to improper use of medication, medication non-adherence, or drug–drug interaction). Thus, CVD medications that decreased the IHD hospitalisation rate could increase it.

## 5. Conclusions

The rate of hospitalisation due to IHD has decreased in England and Wales during the past two decades. This decrease could be explained partly by the increase in the CVD medication prescription rate. Other reasons that likely decreased IRHAs include the decrease in IHD incidence, increase in controlling IHD risk factors, and improved IHD treatments. Future studies exploring other risk factors that are associated with IHD hospitalisation are warranted.

## Figures and Tables

**Table 1:** The percentage of IHD hospital admissions from the total number of admissions per ICD code between 1999 and 2019.

Codes	Indication	Percentage from Total Number of Admissions
I20	Angina pectoris	27.3%
I21	Acute myocardial infarction	29.7%
I22	Subsequent ST elevation (STEMI) and non-ST elevation (NSTEMI) myocardial infarction	3.7%
123	Certain current complications following ST elevation (STEMI) and non-ST elevation (NSTEMI) myocardial infarction (within the 28-day period)	0.1%
I24	Other acute ischemic heart diseases	2.4%
I25	Chronic ischemic heart disease	36.7%

ICD = International Statistical Classification of Diseases system.

**Table 2:** Percentage change in CVD prescription rates from 2004–2019 with a 95% CI.

CVD Medication	Prescriptions Rate in 2004 Prescriptions per 100,000 Persons (95% CI)	Prescriptions Rate in 2019 Prescriptions per 100,000 Persons (95% CI)	% Change from 2004–2019	*p*-Value
Positive Inotropic Drugs	11,028.16 (11,018.49–11,037.82)	7102.62 (7095.15–7110.09)	−35.6%	<0.01
Diuretics	98,042.82 (98,038.55–98,047.10)	76,695.80 (76,683.51–76,708.09)	−21.8%	<0.01
Anti-Arrhythmic Drugs	3540.07 (3534.37–3545.77)	2296.04 (2291.68–2300.39)	−35.1%	<0.001
Beta-Adrenoceptor Blocking Drugs	70,323.27 (70,309.17–70,337.36)	91,063.67 (91,055.38–91,071.96)	29.5%	<0.001
Antihypertensive Therapy	103,520.74 (103,491.01–103,550.47)	170,476.79 (170,442.22–170,511.36)	64.7%	<0.001
Nitrates, Calcium Channel Blockers, and Potassium Activators	82,651.10 (82,624.23–82,677.98)	118,274.89 (118,245.21–118,304.58)	43.1%	<0.001
Sympathomimetic	9.34 (9.04–9.64)	201.74 (200.44–203.05)	2060.4%	<0.01
Anticoagulants And Protamine	15,909.04 (15,897.75–15,920.33)	42,420.62 (42,406.26–42,434.99)	166.6%	<0.001
Antiplatelet Drugs	73,775.65 (73,762.08–73,789.22)	80,284.59 (80,273.02–80,296.15)	8.8%	0.095
Anti-fibrinolytic Drugs and Haemostatics	821.21 (818.43–824.00)	983.75 (980.88–986.62)	19.8%	0.001
Lipid-Regulating Drugs	79,712.64 (79,686.21–79,739.08)	174,784.02 (174,749.11–174,818.93)	119.3%	<0.001
Local Sclerosants	0.90 (0.81–1.00)	0.02 (0.01–0.03)	−98.1%	<0.001

**Table 3:** Correlation between IHD admissions and the prescription of CVD medications between 2004 and 2019.

Table	Positive Inotropic Drugs	Diuretics	Anti-Arrhythmic Drugs	Beta-Adrenoceptor Blocking Drugs	Antihypertensive Therapy	Nitrates, Calcium Channel Blockers, and Potassium Channel Activators	Antiplatelet Drugs	Anti-fibrinolytic Drugs and Haemostatics	Lipid-Regulating Drugs
Angina Pectoris	N/A	N/A	N/A	−0.944 **	−0.855 **	−0.926 **	−0.027	N/A	−0.893 **
Acute Myocardial Infarction	−0.815 *	−0.795 **	−0.684 **	0.838 **	0.448	0.589 *	−0.379	N/A	0.520 *
Subsequent ST Elevation (STEMI) and Non-ST Elevation (NSTEMI) Myocardial Infarction	0.855 **	0.821 **	0.795 **	−0.896 **	−0.621 *	−0.729 **	0.203	−0.718 **	−0.674 **
Certain Current Complications Following ST Elevation (STEMI) and Non-ST Elevation (NSTEMI) Myocardial Infarction (within the 28 Day Period)	0.753 **	0.711 **	0.869 **	−0.789 **	−0.875 **	−0.879 **	−0.234	−0.889 **	−0.874 **
Chronic Ischemic Heart Disease	0.935 **	0.923 **	0.824 **	−0.951 **	−0.578 *	−0.715 **	0.382	N/A	−0.648 **

* *p* < 0.05, ** *p* < 0.01, N/A: not applicable. Bold font highlights that it is statistically significant

## Data Availability

Publicly available datasets were analyzed in this study. This data can be found here: http://http//content. digital.nhs.uk/hes, http://www.infoandstats.wales.nhs.uk/page.cfm?pid=41010&orgid=869 (accessed on 9 June 2021)

## Supplementary Materials

The following are available online at https://www.mdpi.com/article/10.3390/ijerph18137041/s1, Figure S1: Positive inotropic drugs, Figure S2: Diuretics, Figure S3: Anti-arrhythmic drugs, Figure S4: Beta-adrenoceptor blocking drugs, Figure S5: Antihypertensive therapies, Figure S6: Nitrates, Calcium channel blockers and potassium channels activators, Figure S7: Sympathomimetics, Figure S8: Anticoagulants and protamine sulfate, Figure S9: Antiplatelet drugs, Figure S10: Fibrinolytic drugs, Figure S11: Antifibrinolytic drugs and haemostatics, Figure S12: Lipid-regulating drugs, Figure S13: Local sclerosants.
