# Supplementary material for: Hospital Admissions Due to Ischemic Heart Diseases and Prescriptions of Cardiovascular Diseases Medications in England and Wales in the Past Two Decades
Source: Int J Environ Res Public Health. 2021 Jul 1;18(13):7041. doi: 10.3390/ijerph18137041 (PMC8297245; doi:10.3390/ijerph18137041)
Supplement: Supplementary file 1 [file ijerph-18-07041-s001.zip › ijerph-1273999-supplementary.pdf]

Supplementary material:

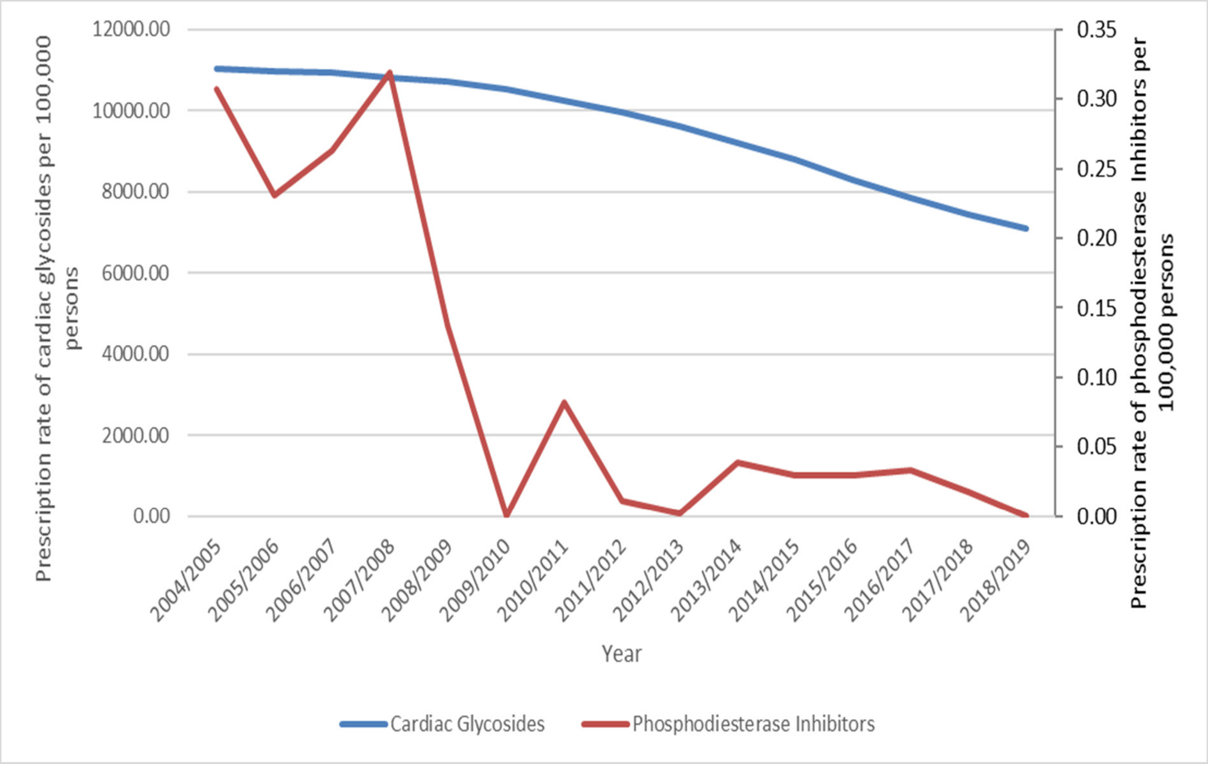

Figure S1: Positive inotropic drugs

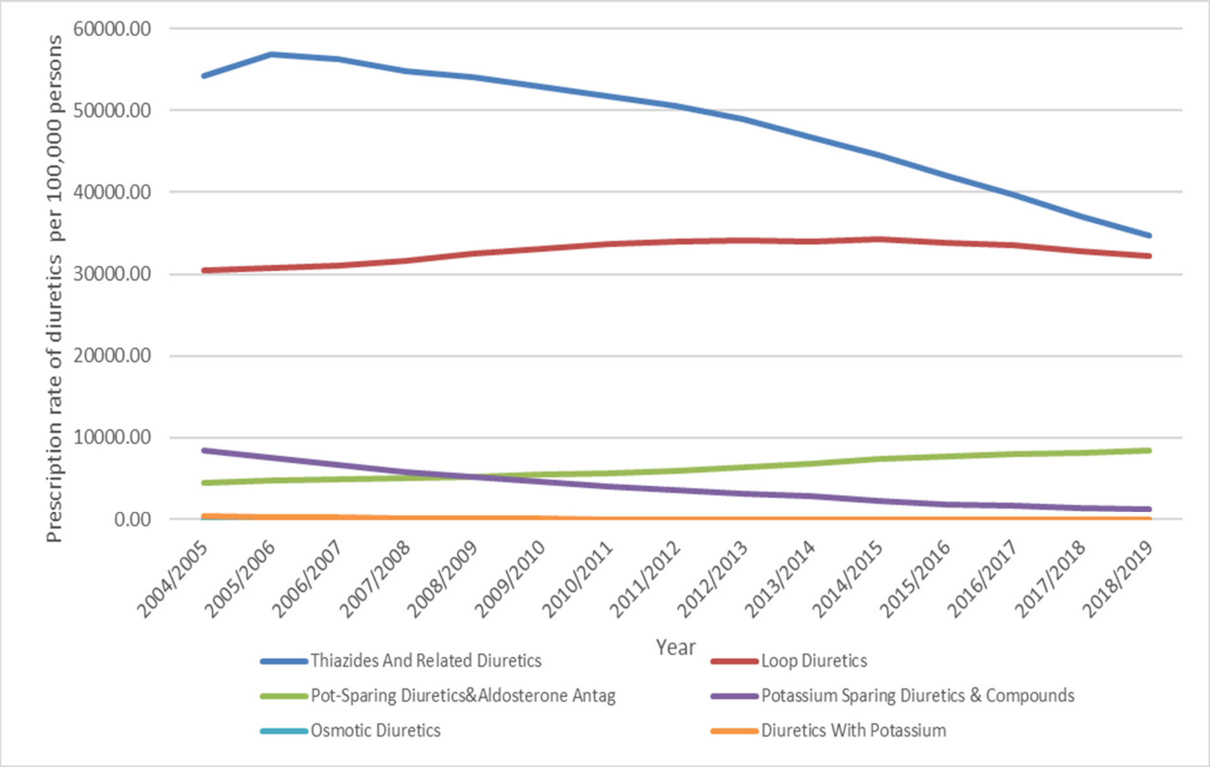

Figure S2: Diuretics

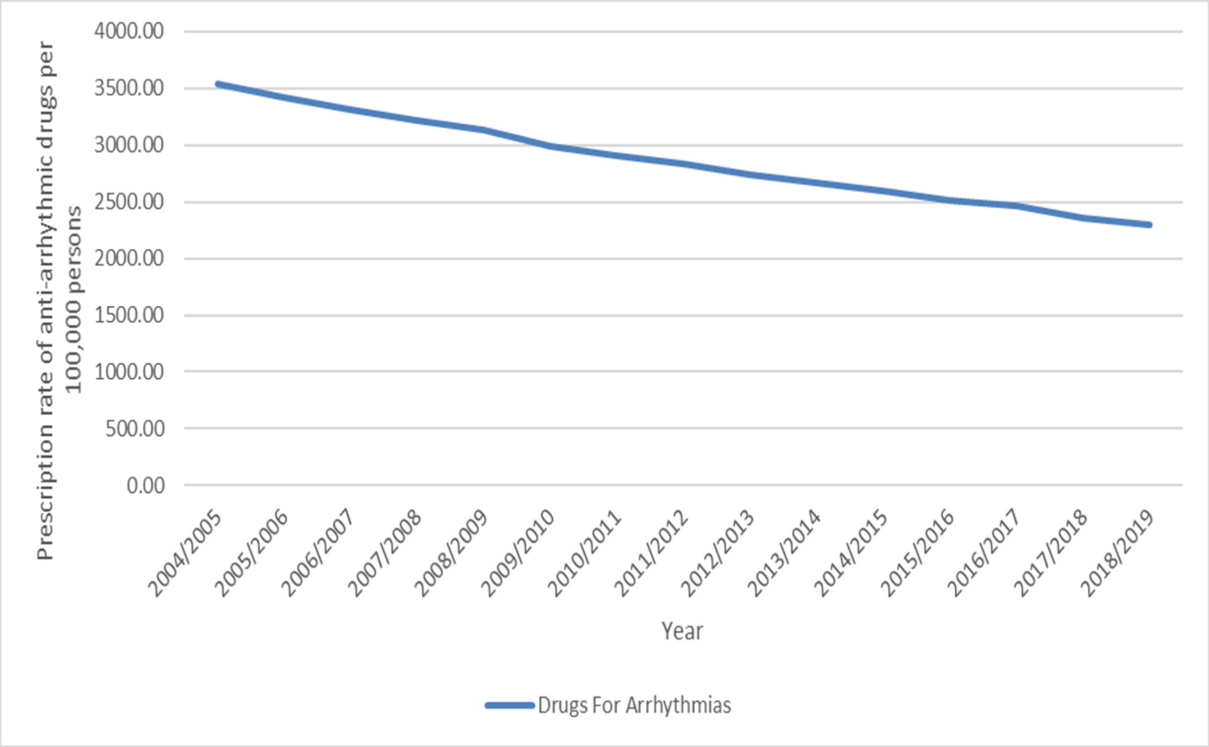

Figure S3: Anti-arrhythmic drugs

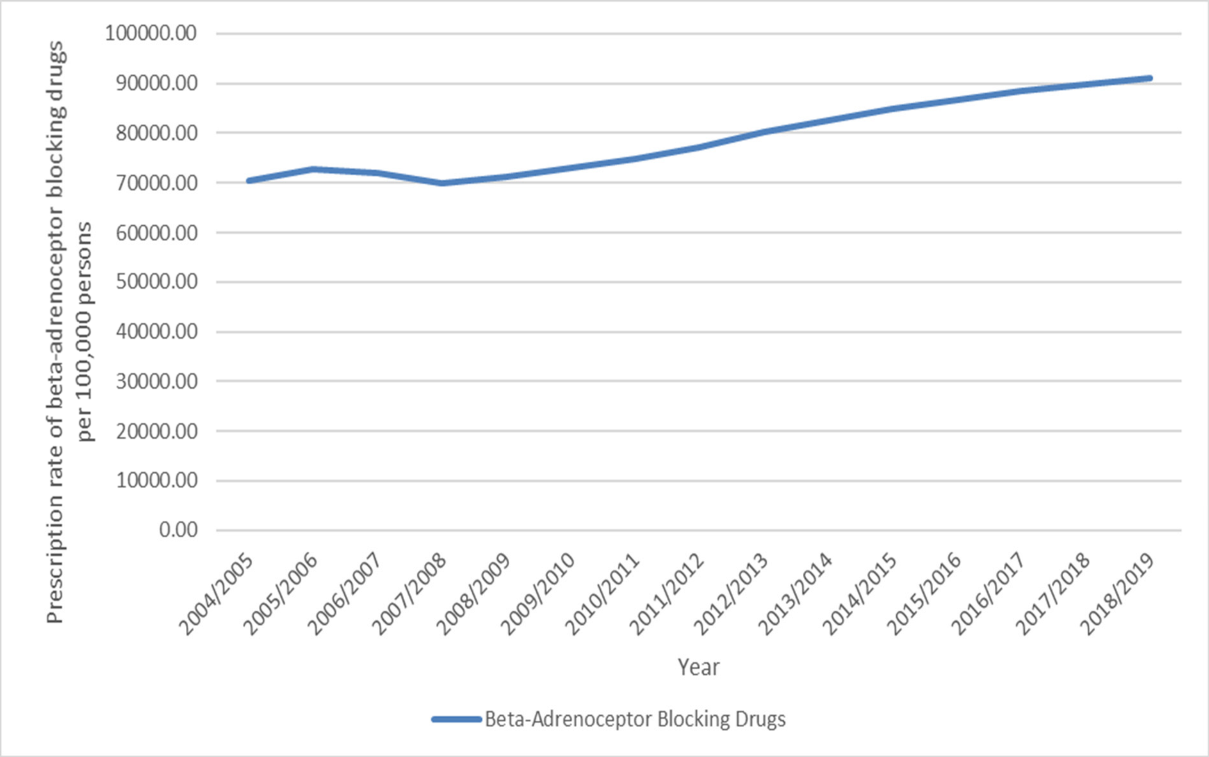

Figure S4: Beta-adrenoceptor blocking drugs

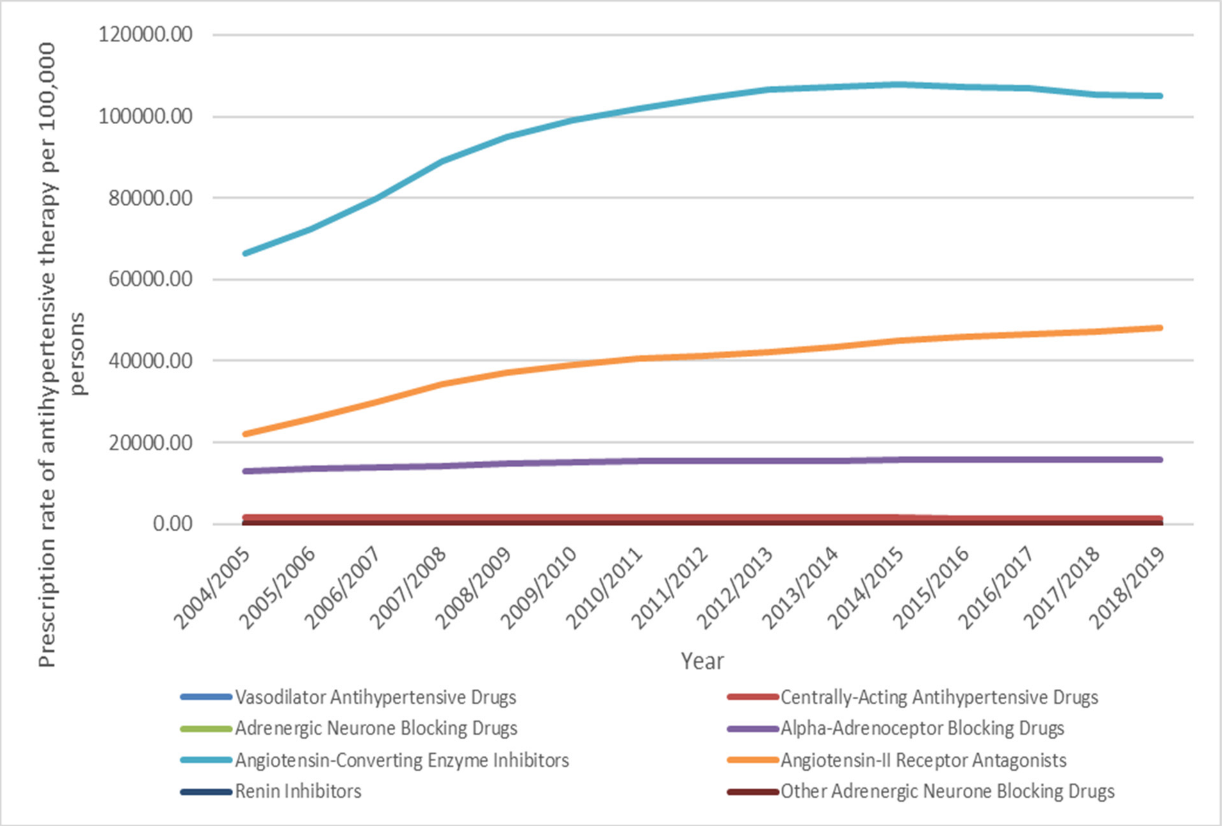

Figure S5: Antihypertensive therapies

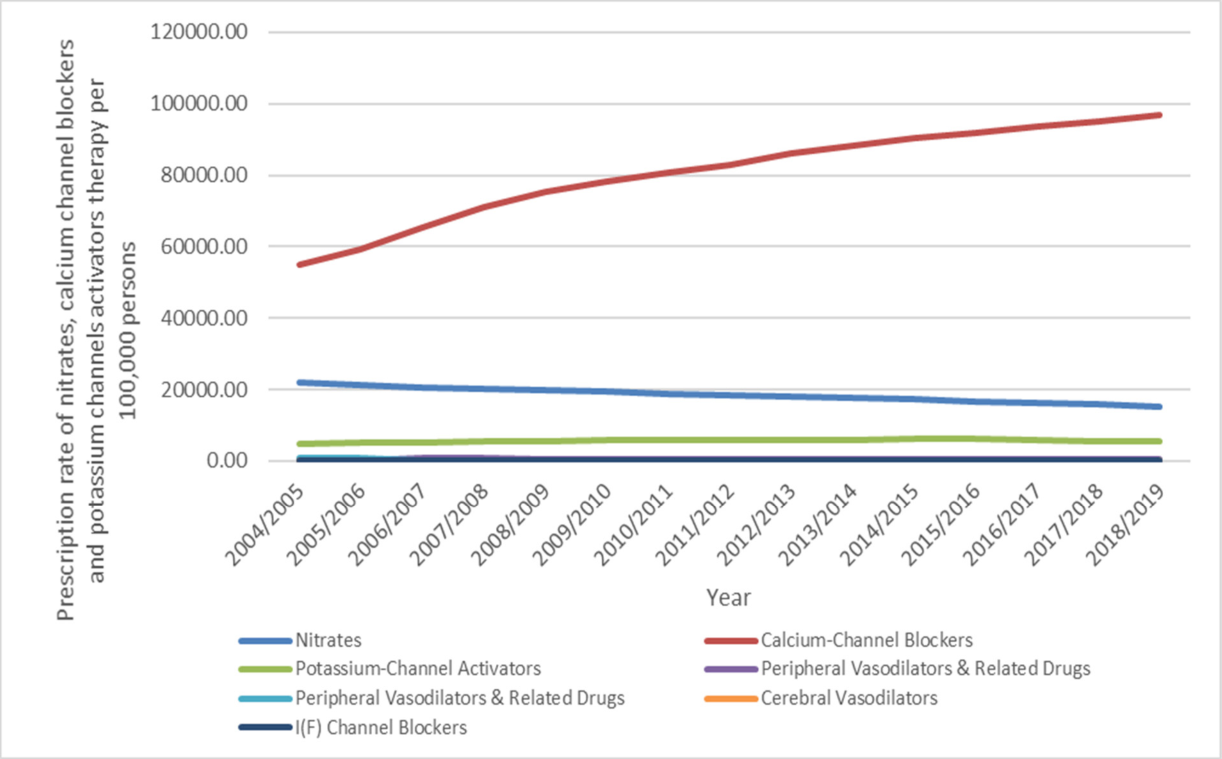

Figure S6: Nitrates, Calcium channel blockers and potassium channels activators

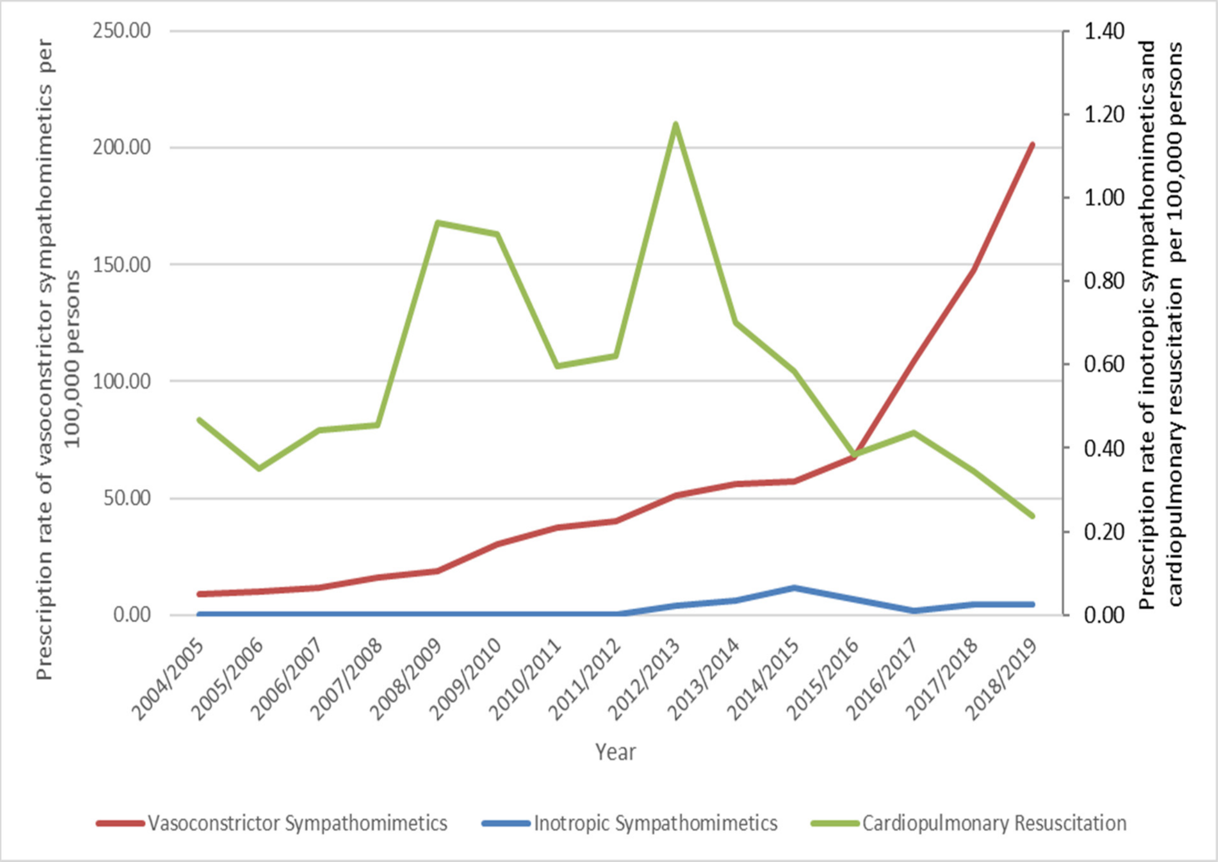

Figure S7: Sympathomimetics

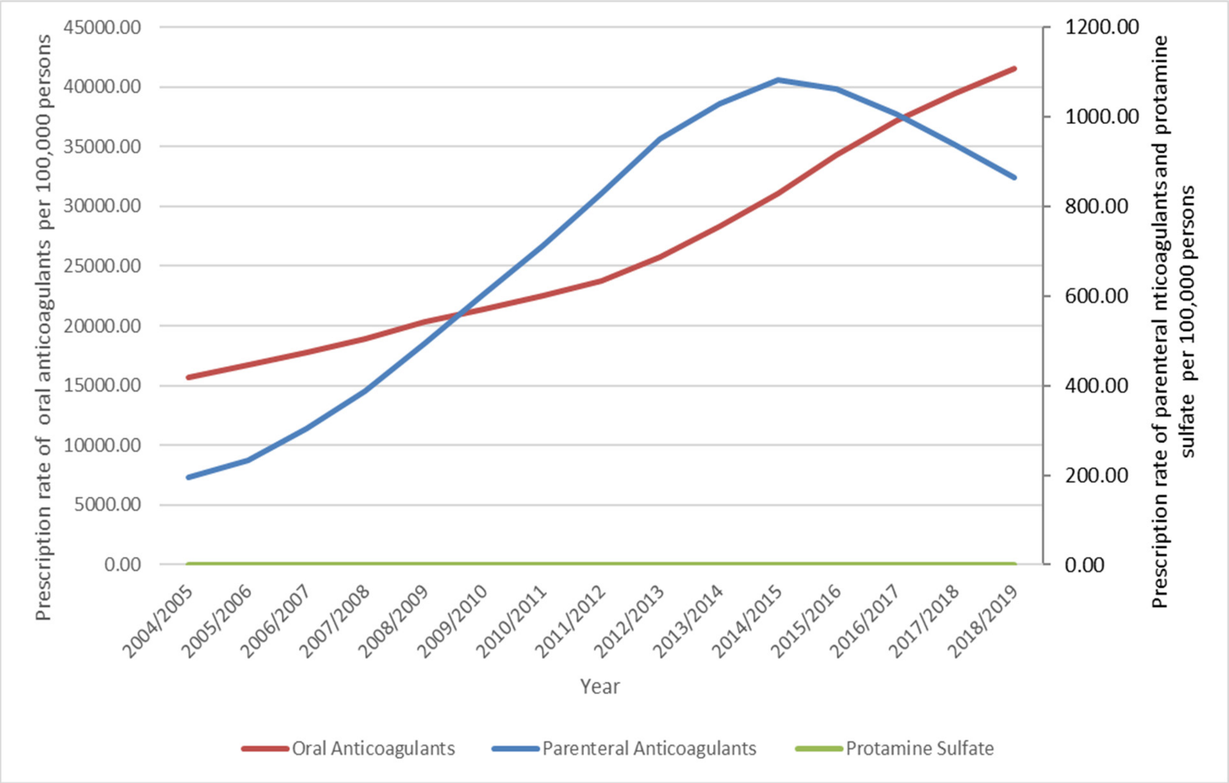

Figure S8: Anticoagulants and protamine sulfate

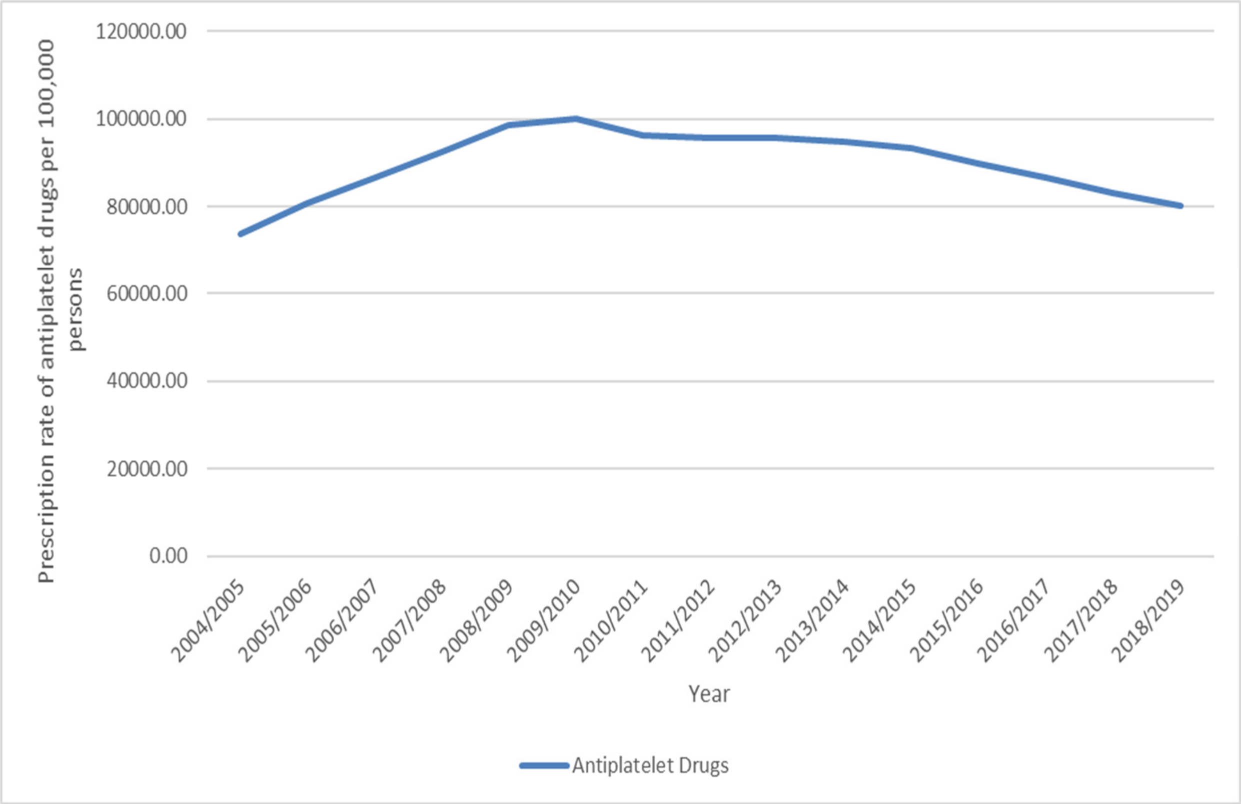

Figure S9: Antiplatelet drugs

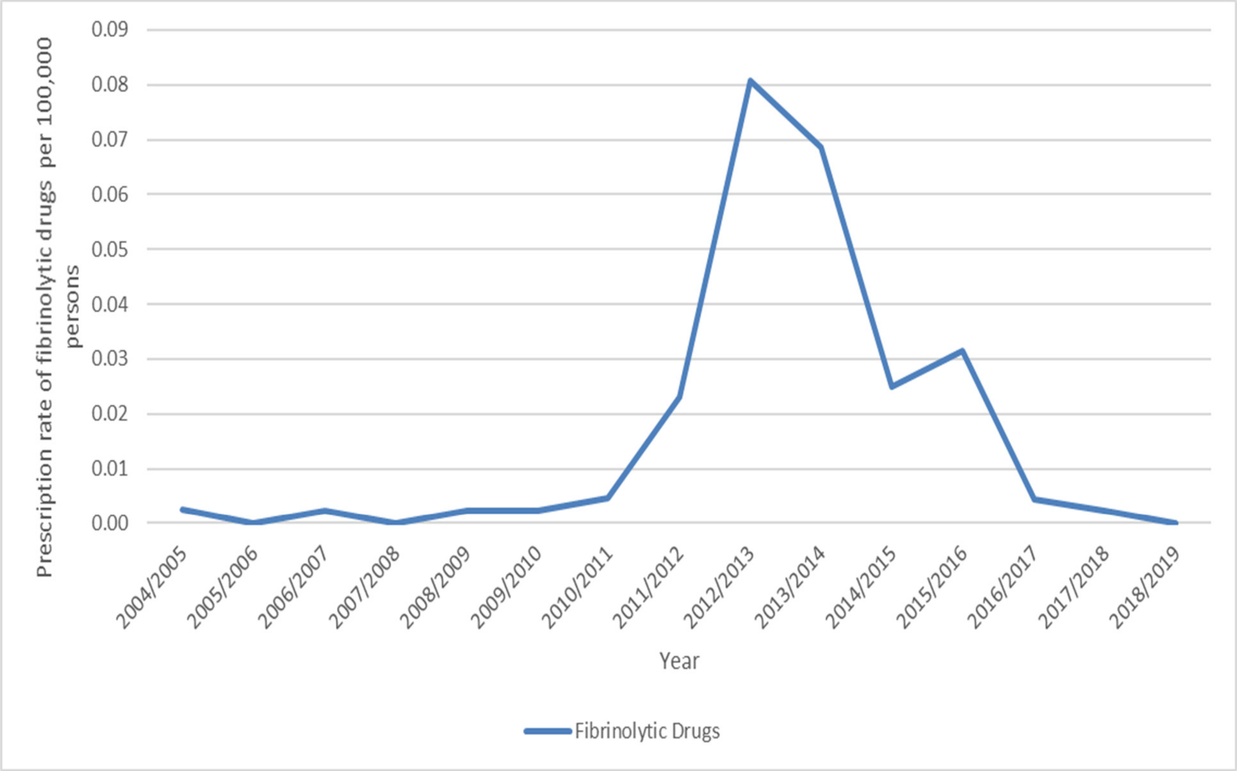

Figure S10: Fibrinolytic drugs

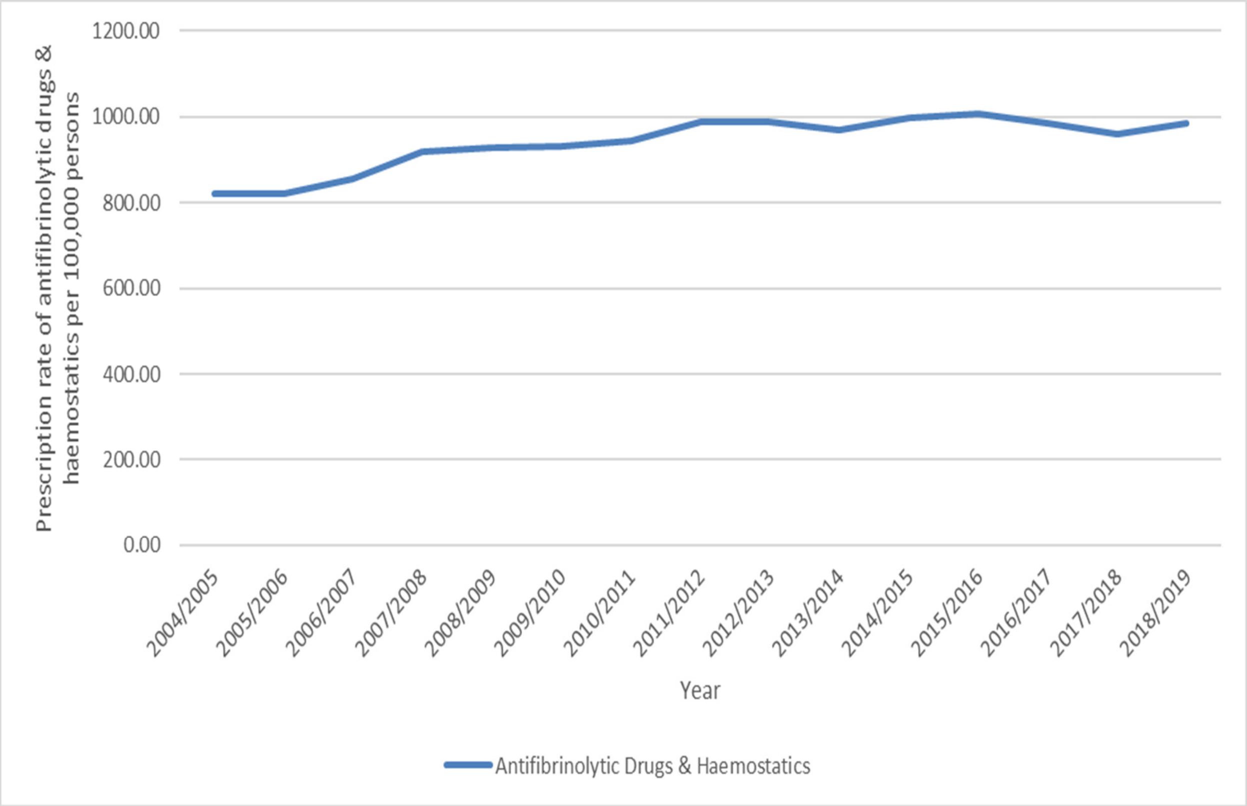

Figure S11: Antifibrinolytic drugs and haemostatics

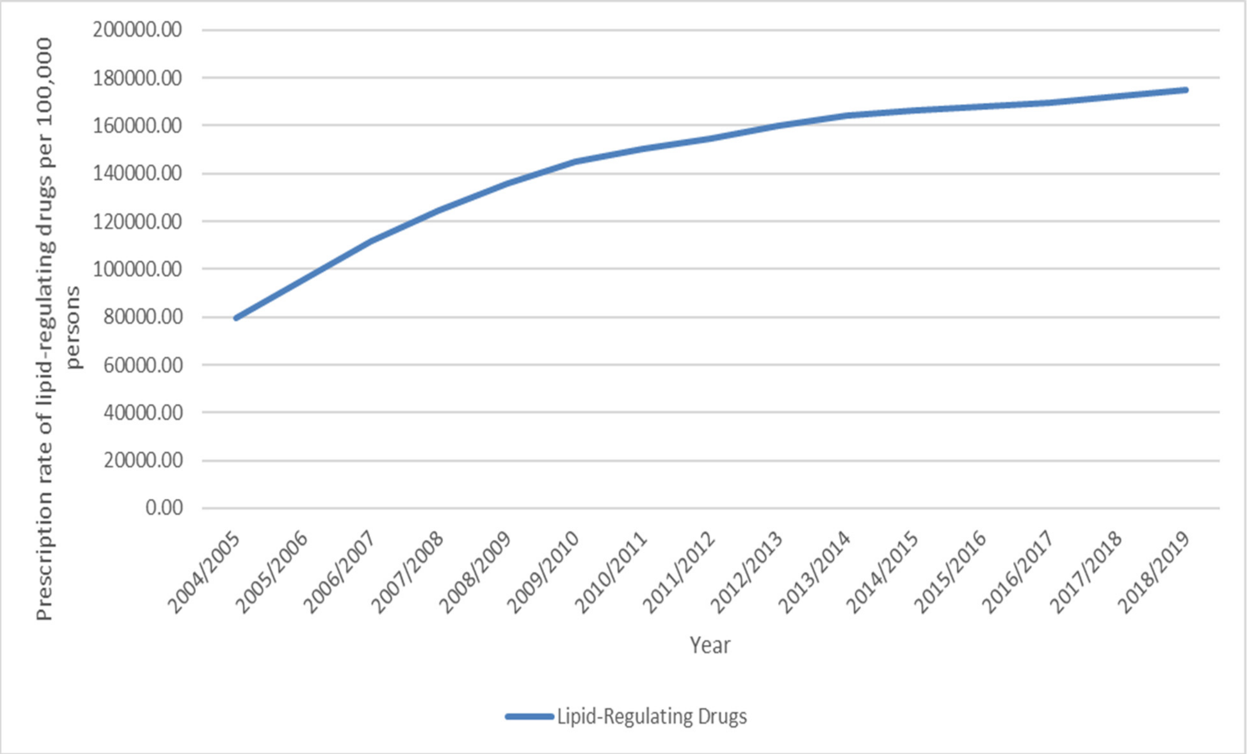

Figure S12: Lipid-regulating drugs

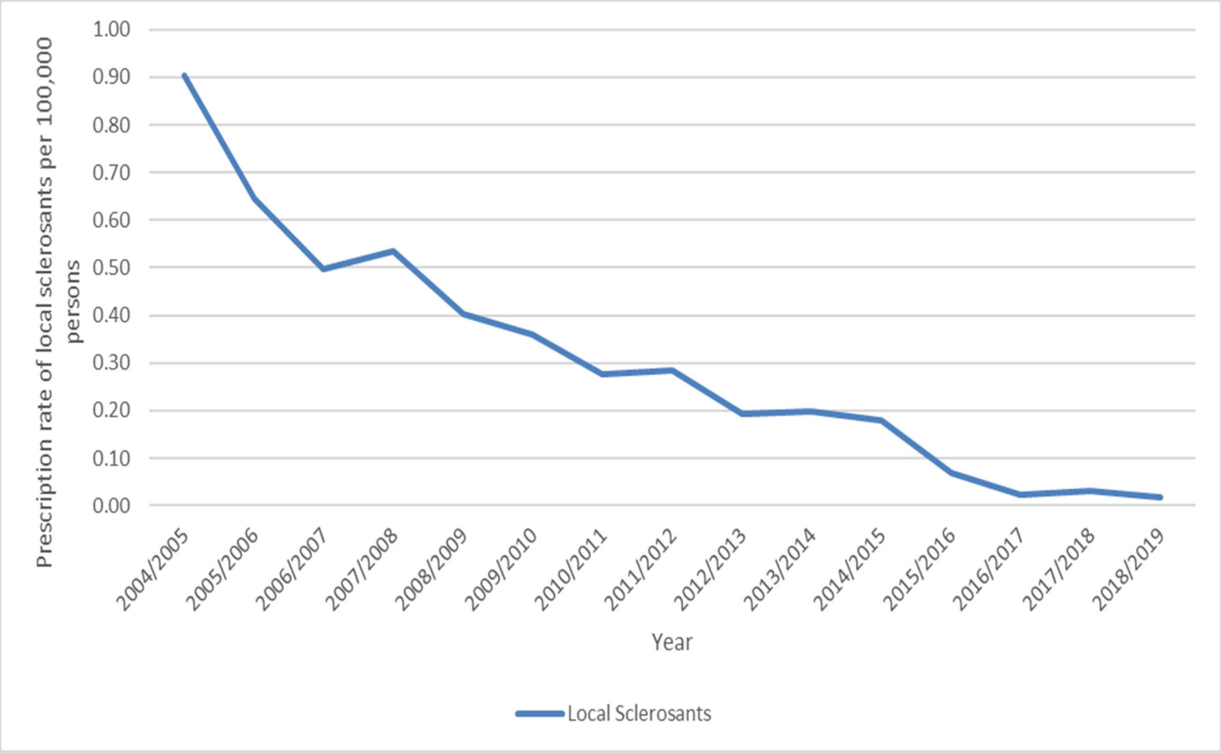

Figure S13: Local sclerosants
